# Radiology-based diagnosis of fungal pulmonary infections in high-risk hematology patients: are we making progress?

**DOI:** 10.1097/QCO.0000000000000937

**Published:** 2023-06-09

**Authors:** Russell E Lewis, Marta Stanzani, Giovanni Morana, Claudia Sassi

**Affiliations:** a Infectious Diseases, Department of Molecular Medicine, University of Padua, Gabelli, Padua; b Hematopoietic Stem Cell Transplantation and Cellular Therapy, Hematology Unit, Regional Hospital Ca’ Foncello, AULSS 2- Marca Trevigiana, Piazza Ospedale; c Department of Radiology, Regional Hospital Ca’ Foncello, AULSS 2- Marca Trevigiana. Piazza Ospedale 1, Treviso; d Pediatric and Adult CardioThoracic and Vascular, Oncohematologic and Emergency Radiology Unit, DIMEC-Dipartimento di Scienze Mediche e Chirurgiche, University of Bologna, IRCCS S. Orsola-Malpighi Hospital, Bologna, Italy

**Keywords:** angiography, computed tomography, invasive aspergillosis, invasive fungal disease, MRI, mucormycosis, PET

## Abstract

In patients with hematological malignancies, high-resolution computed tomography (CT) is the recommended imaging approach for diagnosis, staging and monitoring of invasive fungal disease (IFD) but lacks specificity. We examined the status of current imaging modalities for IFD and possibilities for more effective applications of current technology for improving the specificity of IFD diagnosis.

**Recent findings:**

Although CT imaging recommendations for IFD are largely unchanged in the last 20 years, improvements in CT scanner technology and image processing algorithms now allow for technically adequate examinations at much lower radiation doses. CT pulmonary angiography can improve both the sensitivity and specificity of CT imaging for angioinvasive molds in both neutropenic and nonneutropenic patients, through detection of the vessel occlusion sign (VOS). MRI-based approaches also show promise not only for early detection of small nodules and alveolar hemorrhage but can also be used to detect pulmonary vascular occlusion without radiation and iodinated contrast media. 18F-fluorodeoxyglucose (FDG) PET/computed tomography (FDG-PET/CT) is increasingly used to monitor long-term treatment response for IFD, but could become a more powerful diagnostic tool with the development of fungal-specific antibody imaging tracers.

**Summary:**

High-risk hematology patients have a considerable medical need for more sensitive and specific imaging approaches for IFD. This need may be addressable, in part, by better exploiting recent progress in CT/MRI imaging technology and algorithms to improve the specificity of radiological diagnosis for IFD.

## INTRODUCTION

Fungal pneumonia, primarily caused by *Aspergillus* and less commonly known Mucorales, poses a significant threat to individuals with prolonged neutropenia because of chemotherapy or weakened immune systems following hematopoietic cell transplantation (HCT) [1]. Timely detection of invasive fungal disease (IFD) is crucial in reducing mortality rates as antifungal therapy is most effective early in infection and reduces the risk of dissemination [2]. Although nonculture-based diagnostic tools such as fungal antigen biomarkers and PCR have improved early diagnosis in the last two decades, recommended methods for the diagnostic imaging of fungal pneumonia have remained relatively unchanged. High-resolution computed tomography (CT) of the chest continues to be the preferred diagnostic imaging approach for IFD, with the ability to identify abnormalities in up to 60% of neutropenic patients with fever, even when chest X-rays appear normal [2]. Typical radiological patterns associated with IFD include nodules with or without ground-glass opacities, masses, consolidations, wedge-shaped infarcts, pleural effusions, and in later stages, cavitary lesions [3,4]. These radiologic signs are not specific to fungal infections and can also reflect the presence of co-infections or inflammatory processes. CT findings also play a valuable role in guiding further invasive diagnostic procedures such as flexible bronchoscopy with alveolar lavage and/or CT-guided biopsy, which are essential for establishing a definitive microbiological and histological diagnosis.

Diagnostic criteria for IFD, as described by the European Organization for Research and Treatment of Cancer (EORTC) and Mycoses Study Group Education and Research Consortium (MSGERC) consider CT signs suggestive of IFD in high-risk hematology patients only as indicators of possible IFD [5]. Diagnostic certainty is upgraded to probable IFD in patients who have indirect evidence of IFD such as positive fungal biomarkers, or proven IFD if the patient has direct microbiological or histological evidence of fungal tissue growth/invasion at the site of disease. More recent EORTC/MSGERC diagnostic criteria have expanded the spectrum of radiological patterns for possible IFD based on data suggesting that classic angioinvasive patterns of IFD on chest CT (e.g. nodular infiltrates or masses with or without the halo sign) are less common in nonneutropenic patients treated or patients receiving biological therapies [6^▪▪^]. In these patients, CT findings of airway-invasive disease are more common including, peribronchial densities, centrilobular micronodules, parietal wall thickening of the proximal airways, and tree-in-bud opacities. The higher predominance of airway-invasive versus angioinvasive signs on CT may also explain, in part, the lower sensitivity of the serum galactomannan antigen test in nonneutropenic patients, as galactomannan is shed into the bloodstream during invasion of blood vessels [7,8].

Patients with possible IFD based on host risk factors and CT imaging are a dilemma for clinicians treating an underlying hematological malignancy [9]. These patients have sufficiently high probability (∼40%) for IFD to warrant antifungal therapy, but the optimal antifungal treatment and duration of therapy is unclear in the absence of mycological or histological evidence. A subset of patients with hypoxemia, bleeding and platelet-refractory thrombocytopenia are often too frail for more invasive diagnostic studies such as flexible bronchoscopy or biopsy, or will have procedures postponed to a time when the likelihood of pathogen recovery is lower [10]. Nevertheless, treatment of possible fungal pneumonia often requires postponement of subsequent chemotherapy cycles, HCT, or eligibility for participation in clinical trials, ultimately worsening the prognosis [11]. Patients can also potentially receive weeks to months of unnecessary antifungal therapy that increasingly has severe dose-limiting drug–drug interactions with targeted chemotherapy agents or potentially treatment-limiting nephrotoxicity. These high-risk patients underscore the pressing medical need for novel approaches and technologies that enhance the diagnostic performance of imaging for IFDs.

In this review, we examine the status of current imaging modalities for IFD and possibilities for more effective applications of current technology for improving the specificity of IFD diagnosis. By examining the limitations of current diagnostic imaging approaches, we explore potential avenues for enhancing the specificity of IFD diagnosis through more effective utilization of existing and widely available technologies. 

**Box 1:**
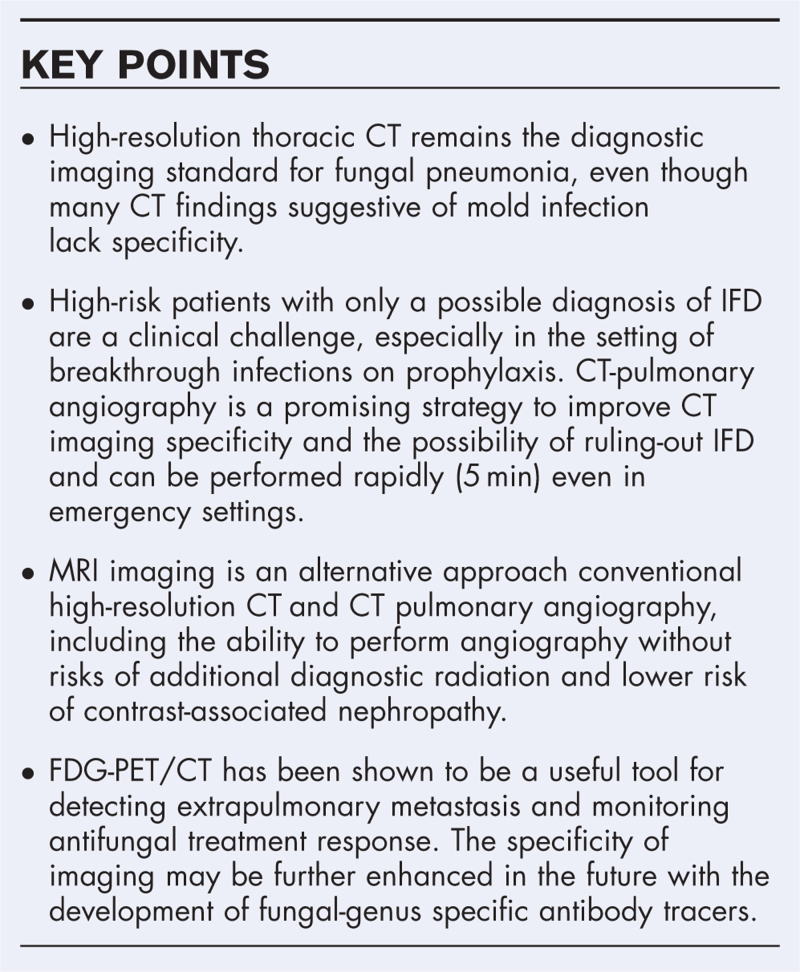
no caption available

## COMPUTER TOMOGRAPHY

Volumetric, high-resolution CT (CT) is the cornerstone diagnostic imaging modality for patients with suspected IFD [6^▪▪^]. CT imaging can identify early signs of fungal infection, such as micronodules (<10 mm), which may appear similar to other parenchymal abnormalities on standard chest X-rays [12]. Despite the continued practice of initially performing standard chest radiography in many centers [13], CT imaging remains indispensable in its capacity to detect subtle manifestations of IFD that may be missed by conventional X-ray techniques.

The most common CT thoracic imaging findings in neutropenic patients with IFD include angioinvasive signs such as small nodular infiltrates with ground glass attenuation (halo sign) or consolidations with air bronchogram. As the infection progresses, larger masses, wedge-shaped infarcts, and pleural effusions may develop. The hypodense sign, characterized by a central hypodensity within a nodule or consolidation, is a highly suggestive, but less frequently observed, finding in patients with IFD undergoing noncontrast-enhanced CT studies [14]. IFD may also present with nonspecific infiltrates, alveolar consolidation, or ground-glass opacities with septal thickening (crazy paving), particularly in patients with preceding viral infections such as H1N1 influenzae or COVID-19 pneumonia [15].

The presence of the halo sign, reverse halo sign, and hypodense signs in CT examinations of patients with neutropenia are highly indicative of IFD. The halo sign is typically observed in over two-thirds of neutropenic patients during initial imaging [16–18] but decreases to less than 40% within 7 days and less than 20% after 14 days [16,19]. This time frame is crucial as patients who receive antifungal therapy when the halo sign is present have higher rates of clinical response and survival compared with patients treated with later CT findings [17].

The atoll or reverse halo sign indicates peripheral hemorrhage surrounding infected lung tissue, but it is much less common than the halo sign. It is seen more frequently in patients with pulmonary mucormycosis compared with aspergillosis [20–23]. However, it can also be present in individuals with sarcoidosis, tuberculosis, pulmonary embolism, and cryptogenic-organizing pneumonia [6^▪▪^]. Chamilos *et al.*[24] reported that the presence of a reverse halo sign with or without multiple (>10) nodules, sinusitis, and pleural effusions are strong indicators of pulmonary mucormycosis.

CT imaging has a crucial role in not only detecting infections early but also in monitoring IFD. It helps in evaluating the size, number, and complications such as vessel erosion and bronchial compression of lesions. In neutropenic patients, nodular lesions linked to pulmonary aspergillosis grow in size despite antifungal treatment. The air-crescent sign and cavitation are a late sign of IFD that may appear when neutrophils remove necrotic debris [25].

A number of studies have suggested that adult, high-risk hematology patients should undergo baseline CT examinations before receiving chemotherapy, as abnormal results on these scans are an independent IFD risk factor [26]. Studies on patients undergoing intensive chemotherapy or HCT have shown that over a third of patients had baseline CT abnormalities, with 10% meeting EORTC/MSG radiographic IFD criteria [5]. Bitterman *et al.* found that more than half of newly diagnosed patients admitted for remission-induction chemotherapy for acute myelogenous leukemia who developed proven or probable IPA during hospitalization had their infections detected by CT scans done before chemotherapy administration [27].

### Low-dose computed tomography

Modern CT scanners and acquisition techniques, along with algorithm-based noise reduction, now enable lung imaging at radiation doses over 90% lower than previously possible (e.g. <0.7 versus 7 mSv) [28,29,30^▪^]. As a result, the risks of diagnostic radiation exposure from repeated imaging are becoming less of a concern and increasingly favor more frequent imaging to reduce the risk of a missed IFD diagnosis. Stanzani *et al.*[30^▪^] investigated the feasibility of performing low-dose (average <0.7 mSv) chest CT within the first 48 h of fever in 68 patients undergoing treatment for hematological malignancies, with a repeat examination within 7 days of follow-up. LD-CT identified radiographic signs of pneumonia in 63% of patients, even though only 39.7% of patients had an abnormal chest X-ray. All patients with CT imaging findings consistent with an IFD diagnosis had positive findings at day 2, albeit at earlier stages (i.e. nodules <10 versus >10 mm) than CTs performed at days 7–10. Earlier detection of lesions shortened the time to bronchoscopy or more specific imaging studies, such as CT pulmonary angiography [31].

### Computed tomography pulmonary angiography

CT pulmonary angiography (CTPA) is the gold standard examination to visualize pulmonary arteries and diagnose pulmonary embolism. The procedure involves injecting an iodinated contrast medium and imaging lungs as the contrast agent flows through the pulmonary arteries. A normal CTPA examination will display a bright white vessel tree indicating patency of pulmonary vessels, while intravascular thrombus or pulmonary embolism will appear as a dark filling defect that interrupts the white contrast-enhanced vessel.

**FIGURE 1:**
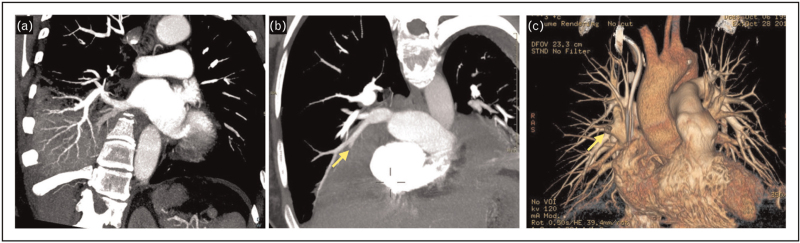
Computed tomography pulmonary angiography for detection of invasive fungal disease. (a) Negative vessel occlusion sign as patent contrast-enhanced vessels are visualized in the right lower lung consolidation of a neutropenic patient with *Pseudomonas aeruginosa* pneumonia; (b) positive vessel occlusion sign (arrow) in the right lung of a neutropenic patient with positive serum galactomannan (probable aspergillosis); (c) volumetric projection of the pulmonary vessel tree demonstrating obstructed vessel (arrow).

Angioinvasive mold infections in the lung infiltrate pulmonary arteries leading to thrombosis and produce a vessel occlusion sign (VOS) similar to pulmonary embolism with interruption of vessel filling inside a circumscribed infiltrate (Fig. 1). The presence of a patent vessel filling inside the suspected infiltrate (absence of VOS) has been correlated to a lack of vessel invasion by fungal hyphae.

Sonnet *et al.*[31] first proposed CTPA for the direct detection of angioinvasive indications in neutropenic patients with IMI. They conducted a study with 10 patients suspected of having IMI and reported a positive VOS in four out of five lesions that were confirmed by histopathology to be IMI, resulting in an 80% sensitivity rate. The only false-negative result was observed in a patient with pulmonary mucormycosis. They did not observe any positive VOS in nine out of nine infiltrates caused by nonfungal pathogens, resulting in a 100% specificity.

Stanzani *et al.*[32,33] compared the diagnostic performance of the VOS identified by CTPA examination with other common radiological signs associated with possible IFD (e.g. halo signs, reverse halo sign, hypodense sign, pleural effusion) in febrile neutropenic patients with hematological malignancies. Although the investigators found that the specificity of a positive VOS was relatively low for probable or proven IFD (51%), this was likely because of the limitations of using a positive serum galactomannan as a criterion for upgrading patients from possible to probable IFD, especially among patients receiving antimold prophylaxis. A positive VOS was detected in 51% of patients with EORTC/MSG-defined possible IFD who did not have positive culture or galactomannan test results. In nearly all cases, where a patient was VOS-positive, the final consensus clinical diagnosis was IFD, while 96% of patients with a negative VOS had an alternative non-IFD diagnosis established by bronchoscopy, culture, or biopsy. Combining cases of proven, probable, and possible IFD adjudicated by final clinical diagnosis, a positive VOS was estimated to be the most sensitive (98%) and specific (89%) CT sign for IFD, with a 100-fold higher estimated diagnostic odds ratio compared with the halo sign (406.40 versus 4.16) [33].

Henzler *et al.*[34] retrospectively analyzed the accuracy of CTPA in diagnosing invasive pulmonary aspergillosis (IPA) in 78 immunocompromised patients with proven or probable IPA compared with 45 matched controls. Their study cohort was unique as only half of the patients had an underlying hematological malignancy, with other common risk factors including solid organ transplantation, solid tumors, and chronic obstructive pulmonary disease. A VOS was found to have the highest diagnostic performance for invasive aspergillosis compared with other radiological signs, with a sensitivity of 94% and specificity of 71%. The frequency of the VOS was not significantly affected by antifungal treatment, and there were four false-positive VOS results in critically ill patients with radiological evidence of invasive aspergillosis and positive serum galactomannan but lacking EORTC/MSGERC-defined host risk factors. Thus, the specificity of VOS was likely underestimated because studied patients did not meet classic host-risk definitions for IFD.

Using CTPA as a routine imaging tool for detecting infections in immunocompromised patients has some limitations because of increased workload for radiologists and the need for expertise in image interpretation. In some cases, a small number of CTPA examinations may be unreadable because of respiratory motion artifacts or technical issues. However, even technically inadequate CTPA studies can provide valuable information, as contrast administration enhances the sensitivity for detecting the hypodense sign. CTPA may not be diagnostic, if the leading vessel is difficult to visualize or for small micronodules (<10 mm) or lesions as the base or apex of the lungs. CTPA also involves additional radiation exposure and the use of iodinated contrast, which may not be suitable for patients at risk for renal dysfunction. The risk of contrast-induced nephropathy (CIN), however, is generally low and can be minimized through careful patient screening and hydration protocols. Several meta-analyses have also reported that actual risk of CIN is much lower than typically estimated by clinicians [35–37]. Therefore, the modest risk of CIN should always be balanced against the consequences of an incomplete diagnostic or interventional work-up caused by avoiding contrast administration.

**FIGURE 2:**
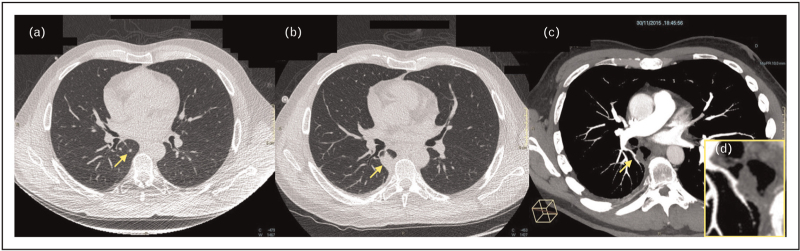
Combined low dose computed tomography (LDCT) imaging with pulmonary angiography to diagnose invasive fungal disease. a 42-year-old man who received alloHCT for acute myeloid leukemia developed Gram-negative *Pseudomonas aeruginosa* sepsis prior to engraftment. Initial LD-CT at the time of initial fever during the first 24 h of neutropenia reveals on axial CT images (a) a small micronodule (<10 mm) in the right lower lung (arrow). Patient developed persistent fever with a repeat LD-CT 10 days later (b) demonstrating enlarged (>10 mm) right lower lobe infiltrate amenable to CTPA pulmonary angiography (arrow). CTPA revealed (c) arterial vessel occlusion (d, inset magnified image) suggestive of angioinvasive fungal disease. Modified with permission from reference [30^▪^]. CT, computed tomography; CTPA, CT pulmonary angiography; HCT, hematopoietic cell transplantation.

The potential for improved early diagnostic specificity for IFD exists through the combined use of LD-CT and CTPA. Stanzani *et al.*[30^▪^] conducted a study in which CTPA was performed on 68 adult hematology patients with fever who had undergone early LD-CT imaging within the first 48 h of fever onset. The VOS was identified in 41% of patients with abnormalities amenable to CTPA examination (39%). This allowed for the confirmation or exclusion of IFD diagnosis in more than one-third of patients within 48 h. For the 60% of patients who were not eligible for early CTPA because of initially positive LD-CT findings, a repeat LD-CT 7 days later identified CT angiography-assessable lesions in an additional 42.8% of patients (see example in Fig. 2). When evaluated versus the final IFD diagnosis status, the VOS was the only CT sign associated with sufficiently low negative likelihood ratio (<0.1) to essentially ‘rule-out’ IFD in a high-risk population of patients [30^▪^].

## MRI

Although MRI is not commonly used to investigate pulmonary diseases because of the high air content in lung tissue and susceptibility to artifacts, it can accurately detect early signs of IFD such as small nodules and alveolar hemorrhage. Thoracic MRI has been found to have similar performance characteristics as high-resolution CT for detecting pneumonia or lung infiltrates in neutropenic patients with acute myeloid leukemia [38,39], and can be used to assess treatment response of malignancy-associated infiltrates or masses with similar sensitivity as 18F-fluorodeoxyglucose (FDG)-PET/CT (FDG-PET/CT) [38]. Pulmonary angiography can also be performed by MRI using T1-shortening contrast agents like gadolinium to produce high-resolution images of the pulmonary arteries and veins [40]. Images can be quickly assessed for perfusion defects and their location caused by angioinvasive fungi, and more detailed postprocessing can provide quantitative assessment of contrast passage kinetics. This allows for the quantification of pulmonary blood flow, volume, and mean transit time, and the generation of parametric maps [40]. Hence, MRI could be a radiation-free alternative to CT and CTPA with lower risk for CIN. However, patients who are short of breath may find it difficult to hold their breath for more than 10 s, and patient positioning and claustrophobia can also lead to scanning difficulties with imaging times up to 20 min, although new ultrafast machines may allow for examinations in less than 5 min. However, MRI imaging is contraindicated in patients with non-MRI-approved metallic devices or pacemakers.

## 18F-FLUORODEOXYGLUCOSE-PET/CT

Although standard CT and CTPA are effective in identifying the location and invasive nature of IMI in the lungs, they have limited sensitivity for detecting extrathoracic IFD and accurate assessments of disease response to antifungal therapy [39,40]. FDG-PET/CT, a whole-body functional imaging technique, has been explored as a tool to assess the metabolic activity of fungal lesions and guide decisions regarding treatment duration or progression to additional chemotherapy cycles. This imaging modality has shown promise in differentiating noninvasive from IPA, monitoring response to antifungal therapy, and detecting extrapulmonary fungal disease [41–43]. Research is also underway to develop antibody fungal tracers that could enhance the specificity of FDG-PET/CT for identifying *Aspergillus* or Mucorales lesions [44,45].

In a study conducted by Douglas *et al.*[46^▪▪^], 147 adult patients with persistent fever (>72 h) receiving conditioning chemotherapy for hematopoietic stem-cell transplantation or chemotherapy for acute leukemia were randomized to imaging work-up with FDG-PET/CT or conventional CT. The primary objective was to compare the effectiveness of the two imaging modalities for rationalizing antimicrobial therapy (defined as appropriately narrowing or broadening therapy) within 96 h of the assigned scan. The results showed that patients who were imaged by FDG-PET/CT were more likely to have rationalized antimicrobial therapy (82 versus 65%, *P* = 0.03) frequently because of narrowing the spectrum of prescribed antibiotics. However, FDG-PET/CT was not significantly better than standard CT in excluding pulmonary fungal infections (82 versus 77%, *P* = 0.51). These findings indicate that although FDG PET/CT may be a promising tool for improving appropriate antibiotic prescribing, there is still little evidence that it can reduce unnecessary antifungal therapy in high-risk neutropenic patients.

## CONCLUSION

In the diagnostic workup of IFD, chest CT is currently the most important test for detecting early infection, staging disease severity, and guiding higher yield invasive testing for microbiologic and histologic diagnosis. However, current CT imaging approaches for IFD lack specificity, making it difficult to fully confirm or rule-out infection. CT pulmonary angiography shows promise in both ruling-in and ruling-out angioinvasive infection characteristics in lung infiltrates, while improvements in MRI lung imaging, if available may provide an alternative to CT, if additional radiation and contrast are contraindicated. FDG-PET/CT imaging is increasingly used to monitor treatment response to antifungal therapy and detect extrapulmonary infection sites. Further research is needed to establish the clinical utility of these less conventional imaging approaches for IFD and their role in improving the specificity of imaging in high-risk patients with hematological malignancies.

## Acknowledgements

*R.E.L. has received research support from Merck & Co, Inc. and Gilead Inc. He also reports speaking fees from Gilead, Pfizer, Avir, F2G and has received compensation for advisory board participation for Gilead, F2G and Cidara Therapeutics. M.S. has received research support from Gilead and support to develop educational materials from Merck & Co.*

### Financial support and sponsorship

*None.*

### Conflicts of interest

*There are no conflicts of interest.*

## REFERENCES AND RECOMMENDED READING

Papers of particular interest, published within the annual period of review, have been highlighted as:

▪ of special interest
▪▪ of outstanding interest
